# What influences decisions about ongoing stroke rehabilitation for patients with pre-existing dementia or cognitive impairment: a qualitative study?

**DOI:** 10.1177/0269215518766406

**Published:** 2018-03-28

**Authors:** Verity Longley, Sarah Peters, Caroline Swarbrick, Audrey Bowen

**Affiliations:** 1 Manchester Academic Health Science Centre (MAHSC), The University of Manchester, Manchester, UK; 2 Division of Neuroscience and Experimental Psychology, The University of Manchester, Manchester, UK; 3 Division of Psychology and Mental Health, The University of Manchester, Manchester, UK; 4 Division of Nursing, Midwifery and Social Work, The University of Manchester, Manchester, UK; 5 Centre for Vascular and Stroke Research, The University of Manchester, Manchester, UK

**Keywords:** Stroke, rehabilitation, decision-making, dementia, cognitive impairment

## Abstract

**Objective:**

To identify factors influencing clinicians decision-making about ongoing stroke rehabilitation for people with pre-existing dementia/cognitive impairment and the impact on clinical practice.

**Design:**

Qualitative semi-structured interviews with stroke specialist healthcare professionals analysed using thematic analysis.

**Setting:**

Acute stroke unit, inpatient stroke rehabilitation units, and community stroke services.

**Participants:**

Twenty three professionals from six multidisciplinary stroke teams involved in decision-making about stroke patients’ rehabilitation potential and clinical pathways.

**Results:**

Factors influencing decision-making about ongoing rehabilitation were (1) gaining understanding of the individual patient, (2) clinician’s knowledge of dementia/cognitive impairment, (3) predicting rehabilitation potential, (4) organizational constraints, and (5) clinician’s perceptions of their role within the team. Decision-making led to two outcomes, either accommodating the pre-existing dementia/cognitive impairment within delivery of rehabilitation or ending rehabilitation for that patient to allocate limited resources where they were perceived more likely to be effective. Participants felt that patients with pre-existing dementia/cognitive impairment had difficulty demonstrating the required rehabilitation potential within the short timescales available in the current model of service delivery. Participants identified a need for training to improve their knowledge and confidence for decision-making and delivery of rehabilitation for this growing population.

**Conclusion:**

Clinicians’ decision-making about ongoing rehabilitation for patients with prestroke dementia/cognitive impairments is influenced by gaps in their knowledge and by service constraints. Increased training and more flexible, patient-centred services would enable clinicians to better accommodate these patients in rehabilitation.

## Introduction

Debates about the suitability of stroke rehabilitation for patients with pre-existing or current cognitive deficits occur regularly in clinical practice and the literature.^1^ An estimated 10% of patients have a diagnosis of dementia prior to first stroke^2^ and others may have undiagnosed cognitive impairment.^3^ Pre-existing dementia/cognitive impairment is associated with poorer functional outcome, discharge to institutional care, and increased risk of death after stroke when compared with those without.^4–6^ It is unclear whether these poorer outcomes are inevitable or are partly a consequence of limited access to stroke rehabilitation. If inadequate rehabilitation is a contributory factor, then that is modifiable through service reorganization. Increasing rehabilitation could improve life after stroke because, although patients with pre-existing dementia/cognitive impairment often start at lower functional baselines, evidence suggests that they benefit from rehabilitation.^7^

UK models of stroke care require professionals to make early predictions about a person’s ‘rehabilitation potential’ for initiating or continuing with rehabilitation.^8,9^ The term rehabilitation potential sits uncomfortably with many but is understood as the ability to benefit from rehabilitation;^10^ a broad process which aims to reduce impairment, increase independence and autonomy, and enhance well-being.^10^ Rehabilitation potential is difficult to predict due to the fact that some patients demonstrate their potential later than others.^10^ Enderby et al.^10^ call for research into decision-making about rehabilitation potential after stroke. It is unclear whether, and if so how, pre-existing dementia/cognitive impairment influences decision-making about rehabilitation potential and treatment plans after stroke.^8,10^

The present research aimed to identify (1) factors influencing the clinicians making decisions about rehabilitation for people with pre-existing dementia/cognitive impairment and (2) how these factors influence clinical practice.

## Methods

The consolidated criteria for reporting qualitative research (COREQ) checklist was used to develop and report this study (see Online Appendix 2),^11^ which was approved by the University of Manchester Ethics Committee (reference no. 16438) and relevant UK National Health Service (NHS) bodies. Clinicians working in stroke services as part of multidisciplinary teams (MDTs) and who were involved in making decisions about rehabilitation were eligible for inclusion. Teams were approached with information about the study, and clinicians volunteered to participate. We sought a purposive sample to include a range of settings and disciplines, for example, hyper acute, rehabilitation, and community in two different NHS trusts, in order to gain as wide a range of perspectives as possible and cover the entire stroke pathway.

One-off individual semi-structured interviews using open and closed questions were undertaken in a private room in the participant’s workplace and followed a topic guide (see Online Appendix 1). The guide was initially piloted on two clinicians working in different services to those sampled from and was reviewed and refined throughout the interview process to ensure it was as relevant as possible. Field notes were made following each interview to aid the topic guide. The choice of face-to-face or telephone interviews were offered to minimize logistical challenges for healthcare professionals.^12^ Informed consent was obtained prior to interview. Interviews were conducted by V.L., an Occupational Therapist (OT) with experience of delivering clinical services to people with stroke and dementia, and of research. This was disclosed to participants prior to interview, and participants were unknown to the interviewer. With consent, interviews were audio recorded and transcribed verbatim by a university-approved transcription service. Transcripts were checked for accuracy prior to deletion of audio recordings.

Data were analysed using thematic analysis.^13^ Analysis started after the first interview and followed constant comparison of each interview with the ones preceding, guiding the point of data saturation.^14^ Data were managed using NVivo 11 software. All identifiable data (e.g. names and places) were removed from the transcripts, which were then read repeatedly in order to increase familiarity. Themes were derived using an iterative process of data familiarization, generation of initial codes, identification of themes, reviewing themes, definition of themes, and report production.^13^ V.L. analysed the transcripts and generated initial codes. A subset of transcripts were analysed by the co-authors, an MDT of health service researchers with expertise in stroke rehabilitation and dementia. Emerging themes were then discussed by all co-authors at each stage of analysis to agree final themes.

## Results

Six multidisciplinary stroke teams across two NHS trusts in the north of England were approached. Twenty three clinicians from six professions volunteered to participate in the study (see Table 1 for demographics). Interviews ranged in length from 15 to 50 minutes (mean = 30, SD = 10.1), and one was conducted via telephone. OTs were most highly represented in the sample (*n* = 11) due to often having the role of assessing cognition in stroke settings. Four physiotherapists, one Speech and Language Therapist (SLT), one Assistant Psychologist, one Clinical Psychologist, three Nurses, and two Physicians were also recruited.

**Table 1.** Summary demographic information of participants.

Characteristics		*N*
Sex	Female	20
	Male	3
Age (years), mean (min–max)	36.25 (22–55)	
Service setting	Hyper acute/acute stroke unit	5
	Inpatient stroke rehabilitation	11
	Community stroke service	7
Years worked in stroke service, mean (min–max)	4 (2 months–12 years)	
Years since qualifying, mean (min–max)	11 (2 years–25 years)	

**Figure 1.**
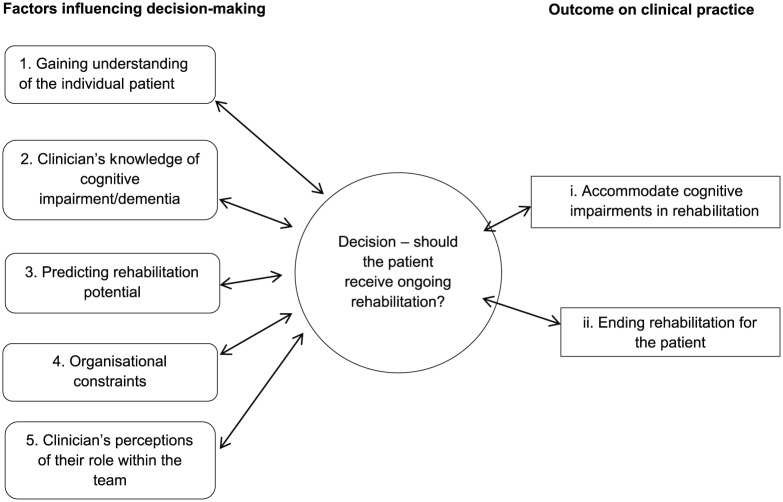
Thematic framework.

Five themes were identified as factors influencing decision-making about rehabilitation with links between them illustrated in Figure 1. Quotes have been aggregated to OT/Physiotherapy, SLT/Psychology, or Nursing/Medical in order to maintain confidentiality.

### Theme 1: gaining understanding of the individual patient

Information gathering on the patient’s prestroke and post-stroke physical and cognitive functioning formed part of the initial assessment by participants and was a key feature in planning rehabilitation. Participants described how several sources of information were used to determine whether a patient had pre-existing dementia/cognitive impairment (see Table 2). Social history from family was perceived to be the most reliable source of information for identifying any pre-existing cognitive issues. Participants spoke of then triangulating this with observations and formal assessments in order to identify prestroke and post-stroke impairments.

**Table 2.** Summary of information sources used to identify pre-existing cognitive impairment.

Type of information	Information source	Example
Formal assessment	Result of assessment during current admission	Functional assessment and cognitive screens
	Results of past assessments	Repeating cognitive screens carried out in the past
Report from others	Social history from family	Asking family/carers whether any impairments are new/old
	Conversation with patient	Asking the patient their previous level of functioning
	Discussion with MDT	Discussing assessments with other colleagues
	Liaison with other services	Contacting GP for history
Other sources	Medical notes	Admission notes/MDT notes, past medical history, repeat admissions
	Gut feelings	They feel more dementia-ry than they do cognitive but I can’t really explain how I get that feel. (P18, OT/Physiotherapy)
	Environmental clues	Observing signs patients are struggling to look after themselves

MDT: multidisciplinary team; OT: Occupational Therapist.

Participants discussed the complexity of identifying cognitive impairments, and the importance of teasing out prestroke and post-stroke impairments in order to identify rehabilitation needs. Participants discussed how patients with existing impairments have different rehabilitation needs to those with new post-stroke cognitive impairments. However, participants from some professions revealed a more nuanced view than others:

We’ve got a lady at the minute that did have dementia before she came in, and [everyone in the team is saying], ‘she’s really confused, she doesn’t have a clue what’s going on … she’s not safe to go home’. Actually I’ve been and assessed her and there’s a lot more cognitive going on than a worsening dementia, like perceptually she has no awareness of her left side. (P16, OT/Physiotherapy, inpatient rehabilitation)

### Theme 2: clinician’s knowledge of dementia and cognitive impairment

Decisions around ongoing rehabilitation for patients with pre-existing dementia/cognitive impairment were influenced by participants’ own knowledge of dementia. While most participants observed that many of the patients they see had pre-existing dementia/cognitive impairments, some were unable to identify patients with dementia:

To be honest, in three months we’ve not really had a real dementia. We’ve had a few mild dementias but not had a proper dementia with a stroke. (P5, OT/Physiotherapy, hyper acute/acute stroke unit)

While this view was not commonplace, it implies that some participants understood dementia as a singular condition rather than a complex one with differing presentations.

In contrast to post-stroke cognitive impairments, participants expressed feeling that they had a lack of knowledge of dementia, which affected their ability to make decisions about ongoing rehabilitation for these patients. Most participants recognized their limited understanding of dementia, attributing this to a lack of training. Instead they relied on ‘common sense’ (P15, OT/Physiotherapy, inpatient rehabilitation) and opportunistic learning:

At uni I think it was quite limited, I’ve learnt most of what I know from cognitive impairment in placements at uni or from work, just shadowing senior staff and things like that. (P3, OT/Physiotherapy, inpatient rehabilitation, 1.3 years in clinical practice)

Participants also highlighted the lack of formal structure and priority available for extra training, especially for ward nurses. Some participants had had specialist dementia training funded by their workplace; however, it was acknowledged that working within stroke services requires a broad spectrum of knowledge, some of which was perceived to be best gained through experience.

### Theme 3: predicting rehabilitation potential

Participants’ knowledge about dementia influenced decision-making about rehabilitation potential. Participants initially described giving all patients the opportunity to have a ‘fair chance’ (P4, OT/Physiotherapy, hyper acute/acute stroke unit) at rehabilitation, but balanced this with their perceptions about the individual patient’s potential to benefit from rehabilitation:

It would be a disservice to say to someone, because you’ve got a dementia you can’t possibly have stroke rehab. (P9, OT/Physiotherapy, hyper acute/acute stroke unit)

Participants discussed their belief that patients need to possess carry-over in order to benefit from rehabilitation: ‘the idea of rehab is that you can build on something and carry over [to the next] session’ (P5, OT/Physiotherapy, hyper acute/acute stroke unit). Carry-over was viewed as an area in which patients with pre-existing dementia/cognitive impairments have difficulty, and participants had lower expectations of the patient’s ability to change:

If there’s pre-existing cognitive impairment there that might be memory related … I would probably then start to think, well what’s this person’s capacity for learning and improving? … the thing we should be doing for that person is discharge planning. (P13, OT/Physiotherapy, hyper acute/acute stroke unit)

While participants expressed the desire to provide fair chances at rehabilitation, these perceptions were associated with a broad belief that having a diagnosis of dementia equated to lack of rehabilitation potential or capacity to change, unless the patient demonstrated otherwise:

I wouldn’t expect anything to improve [if a patient has a pre-existing cognitive impairment] … I just wouldn’t expect [them] to change. (P18, OT/Physiotherapy, community stroke service)

Some also expressed difficulty in determining whether patients possessed rehabilitation potential and were mindful that ‘people have also completely bucked the trend’ (P22, Nursing/Medical, inpatient rehabilitation). Participants described a lack of rehabilitation potential as patients being unable to achieve therapy goals and ‘starting to plateau’ (P1, OT/Physiotherapy, community stroke service) with progress. However, rehabilitation potential was typically assessed within the first few days of admission and participants felt obliged ‘to make a decision pretty quickly and I find that can be quite hard as well’ (P8, OT/Physiotherapy, hyper acute stroke unit).

Previous experience of working with patients with dementia or pre-existing cognitive impairments was used to inform decision-making about rehabilitation potential. Junior participants with less experience in particular highlighted difficulty when determining rehabilitation potential and acknowledged that these decisions held a great deal of weight if they resulted in a patient being discharged into residential care:

But, yeah, I think it’s been so difficult for me to describe who has got rehab potential, it is really difficult. Sometimes it is a gut instinct as well and, yeah, I think it would be good if we did have maybe a bit more guidance on that, you know, what to look out for. (P8, OT/Physiotherapy, hyper acute stroke unit, 3.5 years in clinical practice)

That’s what is scary with the rehab potential part, so if you say this person’s got no rehab potential anymore, then they could essentially have things decided for them … it’s difficult, isn’t it, how long is a piece of string. (P3, OT/Physiotherapy, inpatient rehabilitation, 1.3 years in clinical practice)

Participants also expressed concern that rehabilitation potential is unpredictable. Accurately determining an individual’s rehabilitation potential was viewed as an impossible task and one in which training was lacking:

Nobody’s ever sat down and said, this is how you decide if somebody’s got rehab potential. You kind of get taught that if something’s not working, if you’ve tried it three times and it’s not improving then try something else. (P5, OT/Physiotherapy, hyper acute/acute stroke unit)

### Theme 4: organizational constraints

Participants described how assessing patients with pre-existing dementia/cognitive impairments was challenging and that patients needed increased time to demonstrate progression in rehabilitation which was limited within current service delivery models:

[It is difficult] when we are trying to do our first assessments and then maybe someone with a dementia that’s quite advanced maybe can’t follow instruction, can’t participate with your assessment, maybe is just not very engaged with you … It makes it hard because then you can’t just give them an instruction to follow. (P9, OT/Physiotherapy, hyper acute/acute stroke unit)

Examples were given of how participants worked with patients with pre-existing dementia/cognitive impairment. Participants observed that such patients sometimes required longer to make equivalent progress in rehabilitation than patients without pre-existing impairments:

Rehab as a journey in terms of weeks with people getting better takes longer, but equally individual sessions take longer because you often have to repeat commands, take things really slowly, give people time for delayed processing, so I think it definitely takes more time. (P3, OT/Physiotherapy, inpatient rehabilitation)

Participants described an awareness of management strategies for working with people with dementia, but were struggling to provide these due to service limitations and expressed the opinion that stroke services were not necessarily the most appropriate service for patients with pre-existing dementia/cognitive impairments:

We’re a service that’s very much based around potential to improve, we’re not really a management service and we’ve only got 6 weeks, with those patients I think it’s only fair that we try and get them into the right service. (P1, OT/Physiotherapy, community stroke service)

In addition to this, participants expressed the opinion that the model of rehabilitation they were working within, that is, that rehabilitation led to improvement in function, was not suited to patients with pre-existing dementia/cognitive impairments. It was acknowledged that dementia is a progressive condition that requires a different approach to rehabilitation:

I know a lot of the people with vascular dementia get put on the stroke pathway and it’s actually not the right place for them and they’re not getting the service that they need. They’re also getting a potentially incorrect message in terms of you’re on a rehabilitation ward which means that you’re going to get better, and people with dementias … are potentially going to deteriorate and we can do what we can to support them but we’re not able to rehabilitate them in the sense that they’re going to improve. (P14, SLT/Psychology, inpatient rehabilitation)

### Theme 5: clinician’s perceptions of their role within the team

Participants’ perception of their own role within the team influenced decision-making for patients. Collaborative working was frequently cited as an important factor in decision-making; ‘not just a single thing that we do, we do it as an MDT’ (P10, OT/Physiotherapy, inpatient rehabilitation). Some disciplines perceived a lack of understanding within the team about the scope of their role when working with patients with pre-existing cognitive impairments:

Sometimes they’ll think it’s [our] role to psychologically analyse that person and to provide a full treatment plan for cognitive impairment and to be a psychiatrist and be a psychologist. I think that can be sometimes quite frustrating because they look at us and go, so what we thinking then, do you think they’ve got dementia? And it’s like I’m looking at it in terms of function, do they remember to take their medication, I’m not looking at it to diagnose. (P16, OT/Physiotherapy, inpatient rehabilitation)

Teamwork and gaining specialist knowledge from others was an important factor when making decisions about rehabilitation, and participants used opinions from other disciplines to inform decisions: ‘[I] don’t feel that confident to make that decision on my own at all, and I think it is meant to be an MDT decision as well’ (P8, OT/Physiotherapy, hyper acute/acute stroke unit). This was not without difficulty; some professions sampled perceived their colleagues as having different attitudes about rehabilitation potential to themselves which limited decision-making:

I think us as therapists – physio and OT, we do work obviously very closely on stroke, so it is good because you’re bouncing ideas off each other, but I think [medical staff] can be quite quick to be like, she’s not got rehab potential, they’ve had this stroke and that’s it kind of thing. (P4, OT/Physiotherapy, hyper acute/acute stroke unit)

The previous five themes reveal the factors influencing whether patients would receive ongoing rehabilitation. As shown in Figure 1, these influence decisions about whether to (1) accommodate cognitive impairments into rehabilitation or (2) end rehabilitation for the patient with pre-existing dementia/cognitive impairment.

### Outcome 1: accommodating cognitive impairments in rehabilitation

Participants described focussing on compensatory strategies in order to accommodate patients with pre-existing dementia/cognitive impairments, to maintain function and address safe discharge instead of attempting to improve abilities:

For people where already you’re starting to get a feel that it’s more about a management approach, it’s more about long term potential deterioration rather than improvement. There might be some level of improvement but that will normally be environmental or compensatory rather than doing rehab. (P1, OT/Physiotherapy, community stroke service)

Participants also talked about strategies they used to tailor their approach to rehabilitation for individuals with pre-existing dementia/cognitive impairments (see Table 3).

**Table 3.** Strategies used to support people with pre-existing cognitive impairment or dementia.

Category	Strategy	Illustrative quote
Environmental	• Reduce distractions • Utilize quiet rooms and spaces • Use home visit assessments	I am very conscious of the fact that it’s a very busy, noisy environment and it’s horrendous for a cognitive patient. (P4, OT/Physiotherapy)
Patient-centred approaches	• Spread therapy time throughout the day to minimize fatigue • Ensure patient has eaten, had medication, opened bowels prior to therapy • Use familiar objects during functional assessments • Ensure assessment is meaningful to patient • Engage family with rehabilitation	I’ve done making just a cordial if someone doesn’t make tea because it’s that being meaningful to them, so if someone never made a cup of tea before and I ask them to do it now, it’s just not going to be relevant. (P3, OT/Physiotherapy)
Communication	• Clear, concise instructions • Use closed rather than open questions • Avoid rhetorical questions	You need to be just be asking a yes or no, simple sentence structure, again using really clear, concise language. (P10, OT/Physiotherapy)

OT: Occupational Therapist.

While participants expressed feeling that they had a lack of knowledge and skills for working with patients with pre-existing dementia/cognitive impairment, they actually described a variety of methods that they used to support patients. These were acquired through trial and error and would be reviewed alongside the decision to continue with rehabilitation:

Trying to get strategies to work on [the deficit], so whether that’s compensatory or teaching or equipment or further practice or things like that, … it’s going to be trial and error I think really on your treatment, but trying to think what might be useful to help them overcome that deficit, and then just keep trying until you find something that works or you get to a point where you think, I don’t think this person’s going to be able to get that back. (P3, OT/Physiotherapy, inpatient rehabilitation)

### Outcome 2: ending rehabilitation for the patient

Ultimately, participants described how rehabilitation would have to end for some patients with pre-existing dementia/cognitive impairments. Participants discussed how they would give priority to patients making faster progress in rehabilitation because working with patients with pre-existing dementia/cognitive impairments ‘does add pressure onto the staffing levels’ (P11, Nursing/Medical).

So if I have 18 patients on one ward and I have half of me for that day … if somebody’s got quite significant cognitive problems and I feel that they are not going to make massive difference by […] giving daily therapy, then I will de-prioritise them over … somebody who would benefit from daily input. (P23, SLT/Psychology, hyper acute/acute stroke unit)

Participants discussed how decisions had to be based on outcomes from previous patients in order to facilitate continuing or ending rehabilitation:

When you have such a flow of patients through, all requiring such demanding input … all requiring equal rights and access to this service, there has to be a point when you look at rehab potential and outcome and who was best placed. (P11, Nursing/Medical, inpatient rehabilitation)

Participants talked about service pressures and a reduced ability to provide extensive support when having a large number of patients; treatment became reactive rather than focussed on long-term outcome. This ultimately impacted on how patients who are taking longer to improve would be prioritized within the service. Participants working within time-limited services adapted their rehabilitation to fit within their limits, that is, taking a compensatory approach rather than focussing on improvement within a short timeframe.

I think it’s difficult really and I think sometimes that pressure we’ve got this [six week] window, we know we’ve got the provision to do more but then if we’ve got lots of referrals and we’ve got a big caseload then we’re kind of reduced to what we can do with our patients. (P1, OT/Physiotherapy, community stroke service)

Time limits on services intend to focus interventions and enable prioritization; however, this means that some individuals are not given the opportunity to demonstrate progression within the timeframe. Participants discussed how they felt current stroke pathways were ill-suited for patients with pre-existing dementia/cognitive impairments due to this.

## Discussion

The findings demonstrate the way information, confidence, service models, and team-working inform decision-making about stroke rehabilitation for people with pre-existing dementia/cognitive impairment and their impact on clinical practice. Clinicians attempted to distinguish prestroke from post-stroke cognitive impairments in order to determine rehabilitation needs and potential when working to a somewhat narrow concept of rehabilitation (functional improvement) and towards goals that appeared service-led rather than patient-centred. Often this was based on information from family members and intuition rather than systematic assessment. This identification was influenced by participants’ own knowledge and understanding of dementia, often acknowledged to be limited. Participants expected patients with dementia to have difficulty demonstrating rehabilitation potential, which was confounded by limitations of the model of rehabilitation they were working within. Participants reported patients needed longer to progress with rehabilitation compared to those without prestroke cognitive impairments, but clinicians were required to make early decisions about potential to progress. In addition, misconceptions over roles limited shared decision-making.

The decision of whether a patient will receive ongoing rehabilitation was expressed in two ways. Participants described positive strategies of how they would support patients with pre-existing dementia/cognitive impairments and engaged in an iterative process of reviewing their decision to continue with rehabilitation, shifting their focus from improvement to maintenance. Participants also described how they would have to end rehabilitation for patients with pre-existing dementia/cognitive impairments due to service constraints.

This study has strengths and limitations. A qualitative approach to this topic allowed a relatively unexplored area of clinical practice to be investigated, and this was done from the perspectives of clinicians from a range of relevant disciplines and stroke services. The contextual issues highlighted in this study around working in high pressured environments could be applicable to services nationally. The level of self-reflection from staff demonstrates an awareness of areas for improvement while attempting to work to the best of their current abilities given the imposed service constraints.

This was a difficult topic to discuss in some cases due to sensitivity around sharing working practices caring for vulnerable patients and in an area in which participants felt they lacked skills, knowledge, and resources to provide ideal services for patients. Sampling from two trusts within one geographical area may limit the transferability of the findings; however, the six sampled services were as different as possible and covered a large population. While the sample was more professionally diverse than related studies,^8^ 47% of participants still came from one profession (OT). This was expected due to their role in assessing cognition; however, greater representation from other professions could improve transferability of the findings. The overrepresentation of OTs could underestimate the training needs of this population, because while as a discipline they had received the most training of those sampled and have a role in assessing cognition,^15^ some still lacked confidence. In addition, the fact the lead author (V.L.) is an OT could introduce bias to the study, although every attempt was made to mitigate this. Participant’s awareness of the researcher’s role can affect results; however, the mutual understanding of services may have allowed participants to speak more freely.^16^

While this is to our knowledge the first study exploring decision-making for stroke rehabilitation for people with pre-existing dementia/cognitive impairment, some comparisons to other studies can be drawn. In this study, there was inconsistency over the amount of training and experience participants had about dementia among all disciplines. A lack of knowledge about the aetiology of dementia may impact on the success of interventions in the long term.^17^ Education on dementia has been found to be inadequate for adult nursing, occupational therapy, and social work courses in some UK Higher Education Institutions,^18^ as highlighted by clinicians in this study. Clinical experience has been found to be one of the most important factors influencing decision-making in stroke rehabilitation, therefore training to support those with less experience may be beneficial.^8,9,19,20^ Clinicians in this study indicated their own desire to further develop their knowledge; however, there is limited guidance on working with this patient group, as highlighted in the UK National Clinical Guidelines for Stroke.^21^

Some clinicians in this study saw rehabilitation as an active process leading to improvement in function rather than maintenance of function or well-being; if patients did not demonstrate functional improvement, then they were unable to progress with rehabilitation. This is a somewhat narrow interpretation of rehabilitation, which is defined as restoring, or adapting to loss of, physical and psychological functions.^22^ Adapting to loss could be thought of as maintaining function.^10^ The UK National Clinical Guidelines^21^ even state over time stroke rehabilitation will shift from a restorative to compensatory approach due to the evolving needs for people post-stroke. It seems, therefore, that acknowledgement of different approaches for some patients should be made clear from services delivering rehabilitation. In fact, rehabilitation taking a compensatory approach has been found to be effective for people with dementia^23^ and cognitive rehabilitation can be used to facilitate management of a condition, which is a growing field in dementia care.^24^ In addition, no definitive literature has been identified about specific patient groups who do not benefit from stroke rehabilitation.^25^ Maintenance and management of function remains a vital part of the rehabilitation process, and stroke services need to make provision for people with pre-existing dementia/cognitive impairments who may require more of a management approach.

The current rehabilitation delivery model in the study settings, therefore, appears to have a number of constraints which influence clinical practice. As identified in this study, patients requiring longer to progress in rehabilitation or to demonstrate their potential to change become deprioritized due to limitations around availability of services, which is similar to findings in the wider literature.^8,10,26^ Clinicians working in inpatient environments suggested they were not always conducive to the demonstration of rehabilitation potential for patients with dementia, and therefore, this needs to be considered when deciding if a patient has potential in these settings.^8^ Studies have highlighted challenges in devising meaningful interventions in clinical environments, which particularly impact on patients with cognitive difficulties;^27,28^ and thus, deferring decisions about rehabilitation potential (i.e. deciding when a patient lacks potential) may be more appropriate for these patients.^8^

One aspect to emerge from the study findings is the difficulty clinicians have in judging rehabilitation potential for patients with pre-existing dementia/cognitive impairments. The emotional element of decision-making, with clinicians sometimes fulfilling a need to act or feeling ‘torn’ about seeing patients perceived as a lower priority, has been highlighted in the literature.^8,29^ The concept of giving patients a fair chance at rehabilitation is framed by resource availability and patient abilities, which requires resilience on the part of clinicians.^8,20^ It is clear more support and revised service models are required in order to deliver care that clinicians feel is in patients’ best interests.

Stroke and dementia are associated with age and incidence of both is increasing.^3,30^ Improvements in stroke care mean higher survival rates, and an increase in older patients who are surviving strokes.^31^ Staff in this study identified a lack of training even at university level in working with people with dementia; therefore, it is clear that improvements in education and training are required in order to ensure clinicians possess the appropriate skills to work alongside patients with pre-existing dementia/cognitive impairments, particularly as patient numbers with dementia are likely to increase.^3^

Changes to the current model of stroke rehabilitation are required to better suit the needs of stroke patients with pre-existing dementia/cognitive impairments. Patients are currently required to demonstrate their potential for change early in the acute phase of their treatment; however, this is not always appropriate for this patient group and more flexibility may be required.

Throughout this study, there was little mention of how patients were included in the decision-making process. This could be an area for future study; people with dementia are often excluded from decisions about their care^32^ and exploring how to better involve them in decision-making in the early stages of stroke rehabilitation could potentially facilitate care planning.

**Clinical messages**

- Clinicians should have access to training in order to increase knowledge of dementia and accommodate cognitive problems in rehabilitation.
- Timeframes need to be more flexible for patients to demonstrate rehabilitation potential.

## Supplemental Material

cre-2017-6705-File001 – Supplemental material for What influences decisions about ongoing stroke rehabilitation for patients with pre-existing dementia or cognitive impairment: a qualitative study? Click here for additional data file. Supplemental material, cre-2017-6705-File001 for What influences decisions about ongoing stroke rehabilitation for patients with pre-existing dementia or cognitive impairment: a qualitative study? by Verity Longley, Sarah Peters, Caroline Swarbrick and Audrey Bowen in Clinical Rehabilitation

## References

[1] Lynch EA Luker JA Cadilhac DA et al A qualitative study using the Theoretical Domains Framework to investigate why patients were or were not assessed for rehabilitation after stroke. Clin Rehabil 2017; 31: 966–977. 27421878 10.1177/0269215516658938

[2] Pendlebury ST Rothwell PM. Prevalence, incidence, and factors associated with pre-stroke and post-stroke dementia: a systematic review and meta-analysis. Lancet Neurol 2009; 8: 1006–1018. 19782001 10.1016/S1474-4422(09)70236-4

[3] Prince M Knapp M Guerchet M et al Dementia UK: update. London: Alzheimer’s Society, 2014.

[4] Tatemichi TK Paik M Bagiella E et al Dementia after stroke is a predictor of long-term survival. Stroke 1994; 25: 1915–1919. 8091433 10.1161/01.str.25.10.1915

[5] Appelros P Nydevik I Viitanen M. Poor outcome after first-ever stroke: predictors for death, dependency, and recurrent stroke within the first year. Stroke 2003; 34: 122–126. 12511762 10.1161/01.str.0000047852.05842.3c

[6] Saposnik G Cote R Rochon P et al Care and outcomes in patients with ischemic stroke with and without preexisting dementia. Neurology 2011; 77: 1664–1673. 22042795 10.1212/WNL.0b013e31823648f1

[7] Mizrahi E-H Arad M Adunsky A. Pre-stroke dementia does not affect the post-acute care functional outcome of old patients with ischemic stroke. Geriatr Gerontol Int 2016; 16: 928–933. 26338013 10.1111/ggi.12574

[8] Burton CR Horne M Woodward-Nutt K et al What is rehabilitation potential? Development of a theoretical model through the accounts of healthcare professionals working in stroke rehabilitation services. Disabil Rehabil 2015; 37: 1955–1960. 25495424 10.3109/09638288.2014.991454

[9] Lam Wai Shun P Bottari C Ogourtsova T et al Exploring factors influencing occupational therapists’ perception of patients’ rehabilitation potential after acquired brain injury. Aust Occup Ther J 2017; 64: 149–158. 27654022 10.1111/1440-1630.12327

[10] Enderby P Pandyan A Bowen A et al Accessing rehabilitation after stroke – a guessing game? Disabil Rehabil 2017; 39: 709–713. 27133783 10.3109/09638288.2016.1160448

[11] Tong A Sainsbury P Craig J. Consolidated criteria for reporting qualitative research (COREQ): a 32-item checklist for interviews and focus groups. Int J Qual Health Care 2007; 19: 349–357. 17872937 10.1093/intqhc/mzm042

[12] Sturges JE Hanrahan KJ. Comparing telephone and face-to-face qualitative interviewing: a research note. Qual Res 2004; 4: 107–118.

[13] Braun V Clarke V. Using thematic analysis in psychology. Qual Res Psychol 2006; 3: 77–101.

[14] Boeije H. A purposeful approach to the constant comparative method in the analysis of qualitative interviews. Qual Quant 2002; 36: 391–409.

[15] Intercollegiate Stroke Working Party. Occupational therapy concise guide for stroke 2016. London: Royal College of Physicians, 2016.

[16] Richards H Emslie C. The ‘doctor’ or the ‘girl from the University’? Considering the influence of professional roles on qualitative interviewing. Fam Pract 2000; 17: 71–75. 10673494 10.1093/fampra/17.1.71

[17] Turner S Iliffe S Downs M et al General practitioners’ knowledge, confidence and attitudes in the diagnosis and management of dementia. Age Ageing 2004; 33: 461–467. 15271637 10.1093/ageing/afh140

[18] Pulsford D Hope K Thompson R. Higher education provision for professionals working with people with dementia: a scoping exercise. Nurse Educ Today 2007; 27: 5–13. 16603276 10.1016/j.nedt.2006.02.003

[19] Doyle S Bennett S Gustafsson L. Clinical decision making when addressing upper limb post-stroke sensory impairments. Br J Occup Ther 2013; 76: 254–263.

[20] Luker JA Bernhardt J Grimmer KA et al A qualitative exploration of discharge destination as an outcome or a driver of acute stroke care. BMC Health Serv Res 2014; 14: 193. 24774583 10.1186/1472-6963-14-193 PMC4045916

[21] Intercollegiate Stroke Working Party. National clinical guideline for stroke. 5th ed. London: Royal College of Physicians, 2016.

[22] National Institute for Health and Care Excellence (NICE). Stroke rehabilitation in adults. London: NICE, 2013. 38147522

[23] Graff MJL Vernooij-Dassen MJM Thijssen M et al Community based occupational therapy for patients with dementia and their care givers: randomised controlled trial. BMJ 2006; 333: 1196. 10.1136/bmj.39001.688843.BE PMC1693594 17114212

[24] Clare L. Rehabilitation for people living with dementia: a practical framework of positive support. PLoS Med 2017; 14: e1002245. 28267744 10.1371/journal.pmed.1002245 PMC5340348

[25] Lynch EA Cadilhac DA Luker JA et al Inequities in access to inpatient rehabilitation after stroke: an international scoping review. Top Stroke Rehabil 2017; 24: 619–626. 28835194 10.1080/10749357.2017.1366010

[26] McGlinchey MP Davenport S. Exploring the decision-making process in the delivery of physiotherapy in a stroke unit. Disabil Rehabil 2015; 37: 1277–1284. 25243761 10.3109/09638288.2014.962106

[27] Daniëls R Winding K Borell L. Experiences of occupational therapists in stroke rehabilitation: dilemmas of some occupational therapists in inpatient stroke rehabilitation. Scand J Occup Ther 2002; 9: 167–175.

[28] Whitehead P Fellows K Sprigg N et al Who should have a pre-discharge home assessment visit after a stroke? A qualitative study of occupational therapists’ views. Br J Occup Ther 2014; 77: 384–391.

[29] Doyle SD Bennett S Dudgeon BJ. Sensory impairment after stroke: exploring therapists’ clinical decision making. Can J Occup Ther 2014; 81: 215–225. 29898498 10.1177/0008417414540516

[30] Seshadri S Beiser A Kelly-Hayes M et al The lifetime risk of stroke: estimates from the Framingham study. Stroke 2006; 37: 345–350. 16397184 10.1161/01.STR.0000199613.38911.b2

[31] Feigin VL Forouzanfar MH Krishnamurthi R et al Global and regional burden of stroke during 1990–2010: findings from the Global Burden of Disease Study 2010. Lancet 2014; ccclxxxiii: 245–254. 10.1016/s0140-6736(13)61953-4 PMC4181600 24449944

[32] Kane M Terry G. Dementia 2015: aiming higher to transform lives. London: Alzheimer’s Society, 2015.

