# Supplementary material for: What influences decisions about ongoing stroke rehabilitation for patients with pre-existing dementia or cognitive impairment: a qualitative study?
Source: Clin Rehabil. 2018 Mar 28;32(8):1133–44. doi: 10.1177/0269215518766406 (PMC6068967; doi:10.1177/0269215518766406)
Supplement: cre-2017-6705-File001 – Supplemental material for What influences decisions about ongoing stroke rehabilitation for patients with pre-existing dementia or cognitive impairment: a qualitative study? [file cre-2017-6705-File001.pdf]

Appendix 1: Topic guide

<u>Section 1: Current role</u>

1. Please can you describe your current role?
2. Are you involved in assessing cognition?
    - If so, can you tell me about it?
    - If not, go to question 4.
3. Do you use any specific assessments to assess cognition?
(probe for specifics)
4. What happens if you identify a patient has cognitive impairment?
5. Who raises the suspicions of a cognitive impairment?
6. Where do you get information about a patient's cognition? And where do you record it?
    - i.e. patient notes, liaison with family, through use of assessment
    - What do you do when no family to ask?
7. What's the purpose/your motivation of identifying cognitive impairment?
8. Have you ever had a patient who you suspected had a pre-stroke cognitive impairment? (for example someone with no diagnosis but was struggling at home) Could you describe them? What did you do?
9. How do you differentiate between old and new impairments?
10. How prevalent do you think pre-stroke cognitive impairment is?
11. Is there anything you found difficult working with patients with pre-existing difficulties?

<u>Section 2: Decision making</u>

1. Are there any considerations you make when treating patients with pre-existing cognitive impairment?
2. Would cognitive impairment change the types of intervention you provide?
(probe for example)
3. How do you explain cognitive difficulties to the patient or family?

<u>Section 3: Rehabilitation potential</u>

1. What is it that makes you think a patient will benefit from rehabilitation or not?

2. What do you think other people base decisions about rehab potential on?
    a. And what do you base your decisions on?
3. How do you judge willingness to engage?
4. How do you facilitate motivating patient's to engage?
5. Do you think a patient's cognitive impairment affects length of hospital stay?

Section 4: Knowledge and training

1. Tell me about what, if any, training you have had to support patients with cognitive impairments?

    (Probe for how and by whom it was delivered, content of training, how (if) it's been used within clinical practice)
2. What dementia services are available to you and your patients within the hospital or externally?
    a. What experience have you had of them?
    b. How could they be improved?
3. Is there anything I haven't asked you that you would like to say?

Appendix 2: Consolidated criteria for reporting qualitative studies (COREQ): 32-item checklist

Developed from: Tong A, Sainsbury P, Craig J. Consolidated criteria for reporting qualitative research (COREQ): a 32-item checklist for interviews and focus groups. *International Journal for Quality in Health Care*. 2007. Volume 19, Number 6: pp. 349 – 357

No. Item	Guide questions/description	Reported in section
**Domain 1: Research team and reflexivity**		
Personal Characteristics		
1. Interviewer/facilitator	Which author/s conducted the interview or focus group?	Methods
2. Credentials	What were the researcher's credentials? E.g. PhD, MD	VL – BSc Psychology, BSc Occupational Therapy, PhD candidate
3. Occupation	What was their occupation at the time of the study?	PhD candidate
4. Gender	Was the researcher male or female?	Female
5. Experience and training	What experience or training did the researcher have?	Relevant clinical experience, university training in qualitative methods
Relationship with participants		
6. Relationship established	Was a relationship established prior to study commencement?	Methods
7. Participant knowledge of the interviewer	What did the participants know about the researcher? e.g. personal goals, reasons for doing the research	Methods, Participant Information Sheet
8. Interviewer characteristics	What characteristics were reported about the interviewer/facilitator? e.g. Bias, assumptions, reasons and interests in the research topic	Methods

**Domain 2: study design**		
Theoretical framework		
9. Methodological orientation and Theory	What methodological orientation was stated to underpin the study? e.g. grounded theory, discourse analysis, ethnography, phenomenology, content analysis	Methods
Participant selection		
10. Sampling	How were participants selected? e.g. purposive, convenience, consecutive, snowball	Methods
11. Method of approach	How were participants approached? e.g. face-to-face, telephone, mail, email	Methods
12. Sample size	How many participants were in the study?	Results
13. Non-participation	How many people refused to participate or dropped out? Reasons?	None
Setting		
14. Setting of data collection	Where was the data collected? e.g. home, clinic, workplace	Methods
15. Presence of non-participants	Was anyone else present besides the participants and researchers?	Methods
16. Description of sample	What are the important characteristics of the sample? e.g. demographic data, date	Results
Data collection		
17. Interview guide	Were questions, prompts, guides provided by the authors? Was it pilot tested?	Methods, appendix 1
18. Repeat interviews	Were repeat interviews carried out? If yes, how many?	No
19. Audio/visual recording	Did the research use audio or visual recording to collect the data?	Methods

20. Field notes	Were field notes made during and/or after the interview or focus group?	Methods
21. Duration	What was the duration of the interviews or focus group?	Results
22. Data saturation	Was data saturation discussed?	Methods
23. Transcripts returned	Were transcripts returned to participants for comment and/or correction?	No
**Domain 3: analysis and findings**		
Data analysis		
24. Number of data coders	How many data coders coded the data?	Methods
25. Description of the coding tree	Did authors provide a description of the coding tree?	N/A
26. Derivation of themes	Were themes identified in advance or derived from the data?	Methods
27. Software	What software, if applicable, was used to manage the data?	Methods
28. Participant checking	Did participants provide feedback on the findings?	No
Reporting		
29. Quotations presented	Were participant quotations presented to illustrate the themes/findings? Was each quotation identified? e.g. participant number	Findings
30. Data and findings consistent	Was there consistency between the data presented and the findings?	Yes, findings.
31. Clarity of major themes	Were major themes clearly presented in the findings?	Yes, findings and figure 1.
32. Clarity of minor themes	Is there a description of diverse cases or discussion of minor themes?	Yes, findings.
